# Multi-stage deep learning architecture for carotid artery segmentation and stenosis evaluation: comparative study with digital subtraction angiography

**DOI:** 10.1016/j.jocmr.2026.102683

**Published:** 2026-01-07

**Authors:** Zhiji Zheng, Wanchen Liu, Zhimeng Cui, Hui Fang, Xiao Liu, Kangyi Pan, Qingqing Lu, Kun Zhou, Xiao Luo, Xin Cao, Daoying Geng

**Affiliations:** a Academy for Engineering and Technology, Fudan University, Yangpu District, Shanghai, PR China; b Department of Radiology, Huashan Hospital, Fudan University, Jing’an District, Shanghai, PR China; c Department of Radiology, Shanghai Sixth People’s Hospital Affiliated to Shanghai Jiao Tong University School of Medicine, Shanghai, PR China; d Department of Radiology, the First Affiliated Hospital of Ningbo University, Haishu District, Ningbo, PR China; e Shanghai Engineering Research Center of Intelligent Imaging for Critical Brain Diseases, Jing’an District, Shanghai, PR China; f Institute of Functional and Molecular Medical Imaging, Fudan University, Jing’an District, Shanghai, PR China

**Keywords:** Deep learning, Computer-assisted diagnosis, Carotid atherosclerosis, Arteries stenosis diagnosis

## Abstract

**Background:**

High-resolution magnetic resonance imaging (HR-MRI) provides a non-invasive, radiation-free approach for evaluating stenosis caused by carotid atherosclerosis. However, manual recognition is time-consuming and inter-observer variability. We propose a novel architecture for automated segmentation and stenosis evaluation of extracranial carotid arteries by HR-MRI in comparison with digital subtraction angiography (DSA).

**Methods:**

The 641 stenotic arteries from 422 patients retrospectively collected from three tertiary hospitals were divided into a training-validation set (372 patients, 545 lesions) and an independent test set (50 patients, 96 lesions). An external validation set (89 patients, 168 lesions) was collected from the fourth tertiary hospital.

**Results:**

The architecture demonstrated high consistency with manual segmentation and DSA diagnostic criteria, with mean Dice similarity coefficients of 0.97 ± 0.01, 0.96 ± 0.01, and stenosis evaluation accuracies of 0.88, 0.86 on the independent test and external validation set, respectively.

**Conclusion:**

Thus, the proposed architecture achieved accuracy comparable to manual segmentation by physicians and demonstrated high consistency with DSA diagnostic criteria. By shortening diagnostic time and minimizing inter-observer variability, the proposed architecture is promising to offer a reliable, efficient, and intelligent tool for diagnosing head and neck atherosclerotic disease and assessing stroke risk.

## Graphical abstract

**Figure:**
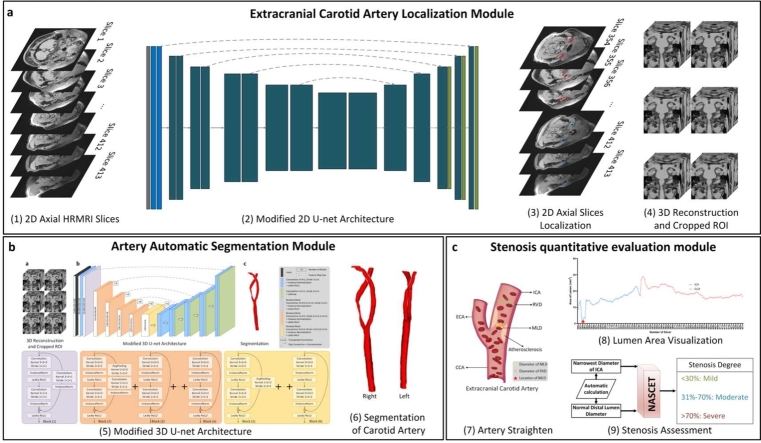

## 1 Introduction

Cerebrovascular diseases, particularly ischemic strokes, remain a significant global health concern. Ischemic strokes account for the majority subtype of stroke worldwide [1], with atherosclerotic disease contributing to one of its leading etiologies, among which carotid artery pathology plays a substantial and well-recognized role in the development of ischemic cerebrovascular events [2], [3]. The risk associated with this systemic and progressive disease primarily hinges on the luminal stenosis and surface irregularities observed at the carotid bifurcation, along with the proximal internal carotid artery (ICA) and common carotid artery (CCA) [4]. Furthermore, the degree of carotid artery stenosis is directly correlated with stroke risk and serves as a critical factor in treatment decision-making, as demonstrated by a large-scale prospective multicenter study [5]. To this end, accurate identification and quantitative assessment of the degree of extracranial carotid artery stenosis are essential for reducing stroke incidence and improving patient outcomes through timely intervention.

Digital subtraction angiography (DSA) has long been considered the “gold standard” for diagnosing carotid artery stenosis due to its high precision [6]. The degree of stenosis is quantified using morphological indicators such as minimum lumen diameter (MLD) and reference vessel diameter (RVD) [7], which are manually measured by physicians via DSA. However, the invasive nature of DSA, along with its reliance on ionizing radiation and the use of contrast agents, raises concerns about potential adverse effects.

In recent years, although magnetic resonance angiography (MRA) techniques such as time-of-flight (TOF) and contrast-enhanced MRA (CE-MRA) have been widely available and commonly used to evaluate lumen stenosis of the artery [8], [9], they exhibit poor visualization of luminal stenosis caused by carotid plaques [10], [11], [12]. Based on this, high-resolution magnetic resonance imaging (HR-MRI) has emerged as a promising candidate for a non-invasive alternative for diagnosing vascular stenosis [13]. It offers superior resolution and contrast for evaluating the artery, providing more comprehensive information than MRA alone. Especially, T1-weighted imaging (T1WI) sequences provide excellent soft-tissue contrast, enabling clear visualization of carotid artery anatomy and identification of stenotic lesions without radiation exposure [14], [15]. It facilitates the early detection of carotid artery abnormalities, which is crucial for timely clinical decision-making [16], [17].

Despite its advantages, manual analysis of HR-MRI images for carotid artery stenosis assessment is time-consuming, labor-intensive [18], and prone to inter-observer variability [19]. These limitations highlight the need for advanced automated tools to improve diagnostic efficiency and accuracy.

Computer-aided diagnosis (CAD) systems, powered by advanced algorithms, have shown significant potential in modern radiology. These systems can automate tasks such as segmentation and diagnosis [20], [21]. CAD systems also assist in tasks such as providing preliminary assessments, flagging anomalies, reducing human error, and improving diagnostic efficiency [22].

Accurate segmentation and quantification of carotid artery stenosis are crucial for early diagnosis and risk assessment of ischemic strokes [23]. Recent advancements in deep learning, particularly U-net architectures and their variants, have shown promising results in carotid artery stenosis assessment on CT angiography scans. For example, Fu et al. [24] proposed an algorithm for rapid segmentation and reconstruction of head and neck vascular images using a 3D convolutional neural network. Based on this, they further developed a deep learning framework that incorporates multiple perspectives to assess stenosis and carotid plaque segmentation using 2D and 3D versions of ResU-Net, respectively [25]. Notably, the use of HR-MRI may further facilitate detailed visualization of the vascular architecture, which is essential for accurate segmentation. Wang et al. [26] proposed a 3D framework for segmenting carotid artery vessel walls from multi-sequence MRIs, and Chen et al. [18] proposed a domain-adaptive and fully automated review workflow, both demonstrating promising results in the segmentation of arteries on MRI. Despite the critical role of HR-MRI in diagnosing carotid artery stenosis, a condition often linked to severe health outcomes such as stroke and other cardiovascular events, few studies have explored fully automated segmentation and quantitative stenosis analysis in this context [27], [28], [29]. Existing segmentation algorithms often fail to adequately account for the complex carotid artery anatomy, including variations in size, bends, and bifurcations, rendering it challenging to maintain a consistent and continuous shape in three-dimensional space. Additionally, the small size of the carotid artery—occupying less than 0.1% of MR image space—compounds the difficulty of accurate segmentation and stenosis diagnosis [26].

To address these challenges, this study proposes a multi-stage deep learning architecture for the automated segmentation and stenosis evaluation of extracranial carotid arteries. By shortening diagnostic time and minimizing inter-observer variability, this architecture is promising to offer significant potential to enhance cardiovascular risk assessment and atherosclerotic disease diagnosis in a non-invasive, radiation-free manner. Its clinical implementation could streamline diagnostic workflows and facilitate the management of carotid artery disease.

## 2 Materials

### 2.1 Patients

As a retrospective study, it has been conducted in accordance with the Declaration of Helsinki (as revised in 2013) and approved by the Institutional Review Board of Huashan Hospital Fudan University (KY2022–647, date: June 6, 2022). The requirement for written informed consent was waived. During the initial selection phase, patients were included based on the inclusion criteria, which were as follows:

1) Patients presenting with clinical symptoms indicative of carotid atherosclerosis, such as ischemic stroke or transient ischemic attacks, supported by relevant imaging studies and clinical data that suggested the presence of carotid artery disease;
2) The examination showed that the intima-media thickness at the bifurcation of the carotid artery and the initial segment of the internal carotid artery was more than 1.5 mm;
3) The patient had undergone complete carotid artery HR-MRI as well as brain MRI.

Patients were excluded according to the following criteria:

(1) Cerebral hemorrhage, brain tumors, or other non-ischemic cerebrovascular diseases identified through cranial CT scans;
(2) Moyamoya disease, severe intracranial artery stenosis or occlusion, arteriovenous malformation, arterial entrapment, aneurysms, or other vascular diseases detected by cranial MRI;
(3) Unilateral or bilateral carotid artery occlusion;
(4) The patients with history of treatment, such as carotid stenting or carotid endarterectomy;
(5) Poor-quality MRI images due to patient movement artifacts or other technical issues.

Accordingly, a total of 511 eligible patient neck HR-MRI scans, acquired between January 2018 and May 2024, were included in the study. These scans were divided into a training-validation set (372 patients, 545 vascular lesions) and an independent test set (50 patients, 96 vascular lesions) by stratified sampling. Additionally, 89 patients with 168 vascular lesions collected prospectively between November 2023 and May 2024 from an independent hospital were treated as an external validation set. All HR-MRI images were converted from the Digital Imaging and Communications in Medicine (DICOM) format to the Neuroimaging Informatics Technology Initiative (NIfTI) format for anonymity and ease of processing[30].

### 2.2 Imaging protocol

#### 2.2.1 MRI data acquisition protocol

All patients included in this study underwent at least one T1WI neck HR-MRI scan before treatment. Imaging was performed simultaneously using an 8-channel phased array carotid coil and a 32-channel combined head and neck coil on Philips Ingenia (Philips Healthcare, Best, the Netherlands), GE Discovery MR750W (GE Healthcare, Waukesha, Wisconsin), and Siemens Magnetom Prisma (Siemens Healthineers, Erlangen, Germany) 3.0T systems, with the patient positioned supine, in a head-first manner on the examination bed. The acquired HR-MRI images were stored in DICOM format. The details of scanning parameters are shown in Table 1.

**Table 1:** Scanning parameters of scanners

		MRI data acquisition protocol	
Manufacturer	Philips Ingenia	GE Discovery MR750W	Siemens Magnetom Prisma
Sequence Names	VISTA	CUBE	SPACE
Imaging Orientation	Coronal	Sagittal	Axial
TR (ms)	600	502	450
TE (ms)	29.32	15.86	13
Flip Angle (^∘^)	90^∘^	90^∘^	120^∘^
FOV (mm^3^)	230 × 161 × 60	240 × 216 × 130	240 × 217 × 176
Thickness (mm)	0.8	1.0	1.0
Acquisition Matrix	368 × 239 × 100	288 × 260 × 130	232 × 256 × 176
Spatial Resolution (mm^3^)	0.6 × 0.6 × 0.6	0.8 × 0.8 × 1.0	0.9 × 0.9 × 1.0

	DSA data acquisition protocol	
Manufacturer	Siemens	GE Medical System
Tube Current (mA)	225	296
Tube Voltage (kV)	64	91
Rotation Angle (^∘^)	200	200
Injection Rate (mL/s)	4	4
Contrast Agent Volume (mL)	20	20
Acquisition Rate (frames/s)	33.25	30
Image Resolution (lp/mm)	3.25	2.5
Matrix Size	1856 × 1856	752 × 750
Voxel (mm^3^)	0.16 × 0.16 × 0.16	0.4 × 0.4 × 0.4

*TR* repetition time, *TE* echo time, *FOV* field of view, *VISTA* Volume ISotropy Turbo spin echo Acquisition, *CUBE* the General Electric version of VISTA/SPACE, *SPACE* Sampling Perfection with Application optimized Contrasts using different flip-angle Evolutions

#### 2.2.2 DSA data acquisition protocol

For the DSA data acquisition, two manufacturers have been utilized: Siemens and GE Medical Systems. After MR imaging, patients underwent 3D rotational DSA within 3–5 days to assess the degree of carotid artery stenosis, which served as the reference standard for validating the results of MR-based segmentation and stenosis evaluation. For all selected patients, anteroposterior, lateral, and oblique projections were acquired. Magnification views were employed when necessary to clarify significant findings. To ensure consistency, all DSA measurements were obtained from equal magnification views and performed with Mitutoyo digital calipers [45]. Proportionality equations were then applied to calculate the normal diameter, stenosed diameter, and percentage stenosis. According to North American Symptomatic Carotid Endarterectomy Trial (NASCET) criteria, the degree of stenosis can be obtained.

It is important to note that DSA is widely recognized in clinical practice as the gold standard for diagnosing clinically significant stenosis, but it does not provide precise quantitative measurements of stenosis in our study. The DSA procedure utilized in this study involved multiple projection views to minimize potential projection bias and provide a more comprehensive assessment of the carotid artery.

### 2.3 Manual delineation

**Fig. 1:**
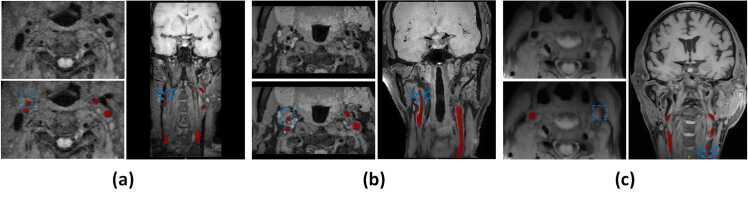
Three types of stenosis degree in our dataset: (a) Mild Stenosis: 0% < the value of NASCET < = 30%; (b) Moderate stenosis: 30% < the value of NASCET < = 70%; (c) Severe stenosis: 70% < the value of NASCET < 100%. *NASCET* North American Symptomatic Carotid Endarterectomy Trial

Manual delineation of the lumen in HR-MRI is a complex and specialized process, particularly when segmenting the extracranial segments of the CCA, ICA, and external carotid artery (ECA) layer by layer. A multi-stage approach ensured accurate segmentation. The initial labeling was performed independently by six radiologists, each with at least two years of radiological experience. Following this, two senior radiologists, with over five years of experience, reviewed the initial labeling, corrected discrepancies, and re-labeled cases as necessary to ensure accuracy. For cases with conflicting labels, a more senior radiologist with over 10 years of experience made the final decision. All labeling results were done using the ITK-SNAP software (V3.8.0, https://www.itksnap.org/). Additionally, the stenosis degree in the extracranial carotid artery was quantitatively evaluated based on the NASCET criteria [31]. Representative examples of carotid artery stenosis within the dataset are presented in Fig. 1.

## 3 Proposed method

**Fig. 2:**
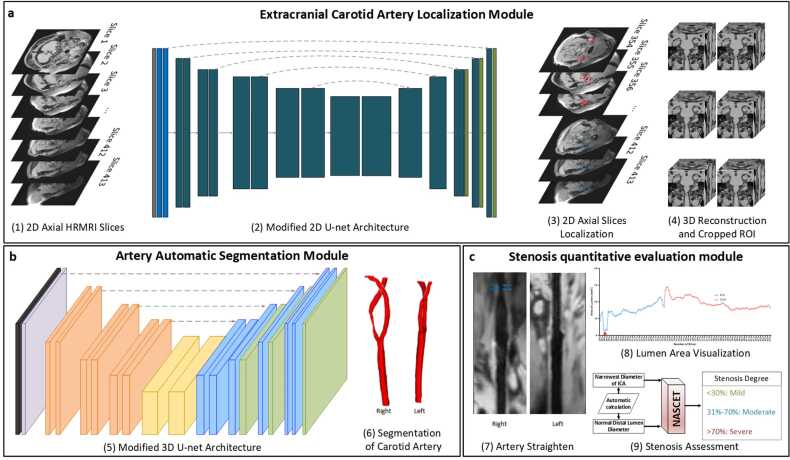
This multi-stage deep learning architecture contains three modules: LC, automatic segmentation module, and stenosis quantitative evaluation module. First, the artery localization module facilitates the localization of ROI from a 2D perspective. Second, the precise segmentation of the carotid arteries from a 3D perspective. Finally, the automatic evaluation of stenosis degree based on NASCET criteria was achieved. *LC* localization model, *ROI* region of interest, *2D* two-dimensional, *3D* three-dimensional, *NASCET* North American Symptomatic Carotid Endarterectomy Trial

In the proposed deep learning-enhanced architecture, three primary modules were developed in sequence as follows: the localization of the ROI, automatic lumen segmentation, and quantitative stenosis evaluation. A comprehensive flowchart for the architecture is presented in Fig. 2.

### 3.1 Image preprocessing and augmentation

Before constructing the carotid artery localization model (LC), the images in the training-validation set were uniformly preprocessed and augmented. During the preprocessing phase, the images were resampled to a consistent voxel spacing of [0.59, 0.46, 0.46], ensuring uniformity across the dataset, which enhanced both model training and generalization. Subsequently, Z-Score-Normalization was employed to normalize the image data by subtracting the mean and dividing it by the standard deviation, thereby ensuring that the data distribution approximated a standard normal distribution.

In the data augmentation phase, inspired by nnU-net [32], we utilized the MONAI library in Python for various augmentation techniques, including rotation, scaling, flipping, Gaussian noise addition, Gaussian smoothing, intensity scaling, and intensity contrast adjustment. For the i-th layer *s*_*i*_, the center of ROI is defined as $\left(\frac{1}{N}\sum_{j=1}^{N}x_{i}^{j},\frac{1}{N}\sum_{j=1}^{N}y_{i}^{j}\right)$, where $x_{i}^{j}$ and $y_{i}^{j}$ are the horizontal and vertical coordinates of the j-th labeled pixel within the *s*_*i*_, respectively. *N* is the total number of labeled pixels. Following that, the inflation radius *r*(*s*_*i*_) of inflated area *s*_*i*_ is defined as equation (1).

(1) $$r\left(s_{i}\right)=\left(1+\epsilon \right)\sqrt{\frac{l*w*N}{\pi }}$$

where *ϵ* is an adjustable factor of inflation and ranges from 0 to 1; *l* and *w* are the length and width of the pixel with dimension $R^{l*w*h}$, respectively.

### 3.2 Extracranial carotid artery localization module

**Fig. 3:**
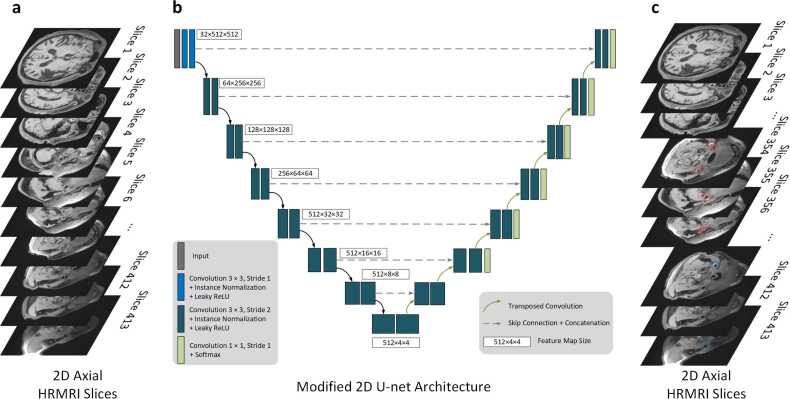
This is the workflow of artery localization module, which consists of an encoder with 8 stages and a decoder with 7 stages. The 2D convolution operation with a normalization and an activation function is performed twice on each stage. Furthermore, the encoder and decode are connected via skip connections. *2D* two-dimensional, *HR-MRI* high resolution magnetic resonance imaging

The proposed carotid artery LC is designed to facilitate the localization of ROI, as illustrated in Fig. 2a. At its core, this module uses a modified 2D U-Net architecture, which consists of an encoder and a decoder, where cross-sectional slices of 3D HR-MRI images are treated as input sequences. The output is the ROI surrounding the extracranial carotid artery, as shown in Fig. 3. This approach narrows the focus for subsequent segmentation of the lumen and stenosis analysis, allowing for more precise evaluation in a smaller region of the carotid arteries, thus improving performance. The details of the network architecture are shown in Supplementary Note 1.

### 3.3 Artery automatic segmentation module

**Fig. 4:**
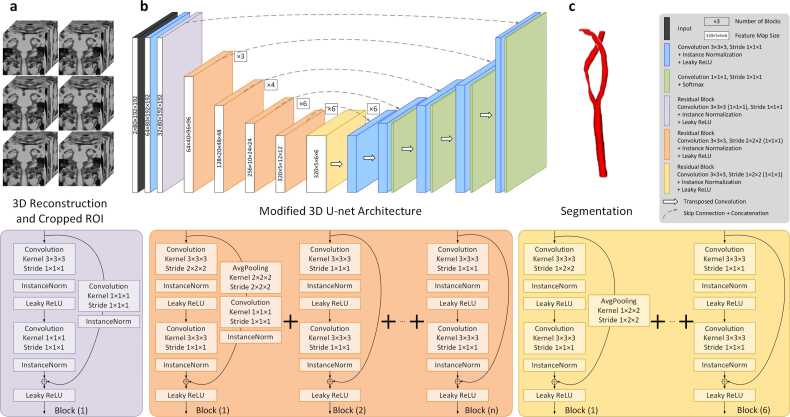
This is the workflow of automatic segmentation module, which consists of a residual encoder with 6 stages and a decoder with 5 stages. The residual blocks, which consisted of 3D convolution operation, normalization, and activation function are performed twice on each stage. Furthermore, the average pooling is employed in the first block of the orange and yellow part. *3D* three-dimensional

As previously described, the slices with ROI outlined by the localization module were reconstructed based on positional information. Subsequently, the reconstructed images were randomly cropped into four patches of 128 × 192 × 192 from each ROI of the carotid artery and employed to train the modified 3D U-Net architecture. To enhance the training efficiency and segmentation accuracy of the model, a residual connection was incorporated into the encoder, as illustrated in Fig. 2b. This design addresses the issue of gradient disappearing and contributes to the overall stability of the deep network, as shown in Fig. 4 [33]. Further details of the modified network architecture are shown in Supplementary Note 2.

### 3.4 Stenosis automatic diagnosis module

As depicted in Fig. 2c, this module comprises three key steps: artery straightening, lumen area visualization, and stenosis assessment.

**Fig. 5:**
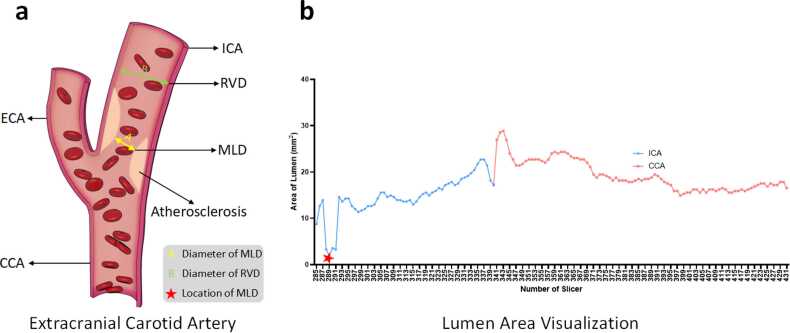
(a) shows the locations of the RVD and the MLD at the stenotic region of the carotid artery. These measurements are essential for determining the degree of stenosis. (b) presents a graph illustrating the lumen area quantification process, where the horizontal axis represents slice number and the vertical axis shows the corresponding lumen area in terms of pixel volume. This approach aids in visualizing the entire extracranial carotid artery and identifying potential stenotic segments. *RVD* reference vessel diameter, *MLD* minimum lumen diameter, *CCA* common carotid artery, *ECA* external carotid artery, *ICA* internal carotid artery

Because of the complicated structure of carotid arteries with twisted or curved arteries, direct measurements of luminal internal diameters can lead to inaccuracies. To address this, the segmentation is straightened to reduce the computational complexity and enhance both the accuracy and reliability of stenosis assessment. This process is accomplished through a newly developed module for the SlicerVMTK extension of 3D Slicer software (V5.4.0, https://download.slicer.org), which reconstructs the three-dimensional artery data based on its geometric centerline. As shown in Fig. 5b, the horizontal and vertical coordinates represent the slice number and the corresponding lumen area (the sum of the pixel volumes), respectively.

The final step involves the quantitative analysis of stenosis degree, performed on straightened arteries. Specifically, the luminal internal diameter at the dilated portion of the carotid artery and the narrowest portion of the ICA serves as the RVD and MLD, respectively, as shown in Fig. 5a. The details of this module as shown in Supplementary Note 3.

## 4 Results

### 4.1 Patient characteristics

A total of 511 patient-wise datasets from four tertiary hospitals, encompassing 809 diseased vessels, were included in the study cohort. These datasets collected from three tertiary hospitals between January 2018 and October 2023 were divided into a training-validation set (372 patients with 3 mild, 206 moderate, and 336 severe lesions), an independent test set (50 patients with 4 mild, 20 moderate, and 72 severe lesions). An external validation set was collected prospectively between November 2023 and May 2024 from the fourth tertiary hospital (89 patients with 6 mild, 42 moderate, and 120 severe lesions). This retrospective, multicenter study was approved by the hospital’s institutional review boards (KY2022–647).

Given the small number of patients with mild carotid stenosis and the significantly lower risk of stroke in patients with mild stenosis compared with severe stenosis (>70%)[38], mild and moderate lesions were combined for comparison with severe carotid stenosis ones, reducing the classification task from three categories to two. The demographic characteristics and lesion characteristics are summarized in Table 2.

**Table 2:** Demographic information and lesion characteristics of patients

	Training-validation			Independent test			External validation
	Center I	Center II	Center III	Center I	Center II	Center III	Center IV
*Patient-wise*							
No. of patients (n)	183	61	128	28	9	13	89
No. of male patients (n)	134	47	109	19	7	11	78
No. of female patients (n)	49	14	19	9	2	2	11
Mean age (mean ± SD)	65.21 ± 9.23	67.16 ± 7.54	64.84 ± 7.97	66.39 ± 8.65	64.56 ± 9.23	67.08 ± 10.77	65.30 ± 9.16
Male patients (mean ± SD)	65.81 ± 9.25	66.81 ± 7.76	65.09 ± 7.85	68.42 ± 8.60	63.71 ± 8.12	67.18 ± 11.77	65.71 ± 8.76
Female patients (mean ± SD)	63.57 ± 9.06	68.36 ± 6.89	63.40 ± 8.69	62.11 ± 7.46	67.50 ± 16.26	66.50 ± 2.12	62.45 ± 11.74
Days btwn MRI & DSA (mean ± SD)	2.97 ± 1.46	3.82 ± 1.53	2.66 ± 1.57	3.07 ± 1.46	3.89 ± 1.45	3.08 ± 2.02	3.03 ± 1.46
ScI (n)	-	15	97	-	2	9	13
ScII (n)	110	31	31	16	4	4	12
ScIII (n)	73	15	-	12	3	-	64
*Lesion-wise*							
No. of diseased vessels (n)	265	89	191	55	16	25	168
Degree of carotid stenosis:							
0% < MildStenosis≤30% (n)	2	-	1	2	-	2	6
31%≤ModerateStenosis≤70% (n)	95	47	64	9	5	6	42
71%≤SevereStenosis < 100% (n)	168	42	126	44	11	17	120

*n* number, *mean* mean value, *sd* standard deviation, - donated as null value. Moreover, ‘ScI’, ‘ScII’, and ‘ScIII’ refer to the Philips Ingenia (ScI), GE Discovery MR750W (ScII), and Siemens Magnetom Prisma (ScIII) acquisition platf, respectively

### 4.2 Implementation details

**(1) Training schedule:** The proposed deep learning-enhanced architecture was trained as an entirety and performed five-fold cross-validation. In each fold, the training-validation set was randomly partitioned into four groups for training, with the remaining group as validation set, using the scikit-learn library (V1.2.1, https://scikit-learn.org/). Given the inclusion of both 2D and 3D models within the framework, variations exist in the specific training parameters. Specifically, the batch size for the 2D one was set to 12 and trained for 1000 epochs with a variable learning rate. The initial learning rate was set to 0.01, and it varied according to the following formula: ${\left(1-\frac{{epoch}_{c}}{1000}\right)}^{0.9}$ denotes current epoch [34]. For the 3D model, the batch size was set to four and had the same training epochs and learning rate as the 2D one. The optimizer employed stochastic gradient descent (SGD) with a Nesterov momentum (*μ*) set to 0.99. To further enhance training efficiency, deep supervision was also incorporated. The loss function combined with the binary cross-entropy loss and the dice were employed in the training schedule with equal weighting (1:1 ratio). The training schedule and all subsequent experiments were realized using MONAI (V0.9.1, https://docs.monai.io), a Python framework for medical image segmentation. The computations were performed on a system with the following specifications: CPU, Intel Core i9–14900K, RAM, 32 GB, and GPU, NVIDIA GeForce RTX 4090D 24 GB.

**(2) Quantitative metrics:** To comprehensively quantify the performance of the proposed deep learning-enhanced architecture, the evaluation metrics used in the experiments are detailed in Supplementary Note 4. The metrics evaluate the segmentation results of the extracranial arteries and the lesion-wise diagnostic performance.

The agreements of the volumes between the segmentation results and ground truths were calculated using the concordance correlation coefficient (CCC) [35].

**(3) Ablation and benchmark experiments:** In the ablation study, the effectiveness of the LC module was assessed by removing it and directly inputting the image into the segmentation module. This modification enabled us to evaluate the contribution of the LC module to the overall model performance. For the benchmark experiments, 3D-UNet [36] and U2Net [37] were used as backbone models to replace the proposed localization and automatic segmentation modules. These experiments served as baselines for comparing the performance of our proposed model. In both the ablation study and benchmark experiments, the preprocessing steps, training schedule, and fold-ensemble methodology were kept consistent with those used in the proposed model.

**(4) Statistical analyses:** To statistically analyze the results of all experiments, R software (V3.6.2, https://r-project.org) and Python (V3.8.16, https://python.org) were used. Initially, patient-wise demographics and lesion-wise characteristics were statistically analyzed, and the training-validation set and independent test set were assigned in the mean of stratified sampling by R software sequentially. Following that, the segmentation results of patient-wise were analyzed at both the segmentation level and detection level using MONAI library. For the independent test set and external validation test, the differences in the performance were calculated using Wilcoxon signed-rank test. Furthermore, per artery was treated as a diagnostic subject to calculate accuracy (Acc), F1-Score, Sens, precision, Spec, and area under the curve (AUC). Finally, lesion-wise analysis was conducted, including correlation plotting and the calculation of CCC. Notably, statistically significant was defined as P < 0.05.

### 4.3 Performance of carotid artery segmentation

**Fig. 6:**
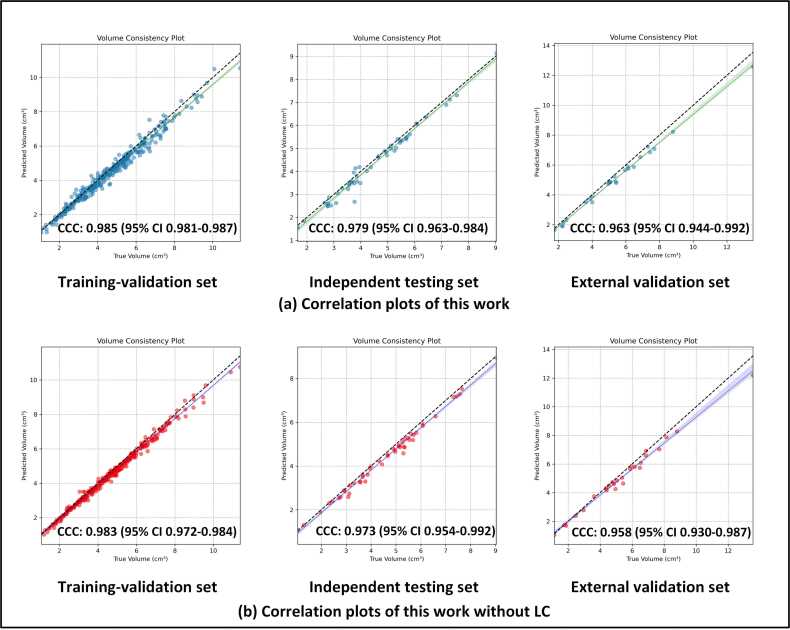
This figure presents the correlation plots for segmentation performance analysis in the training-validation set, the independent test set, and the external validation set. *CI* confidence interval, *CCC* concordance correlation coefficient

**Fig. 7:**
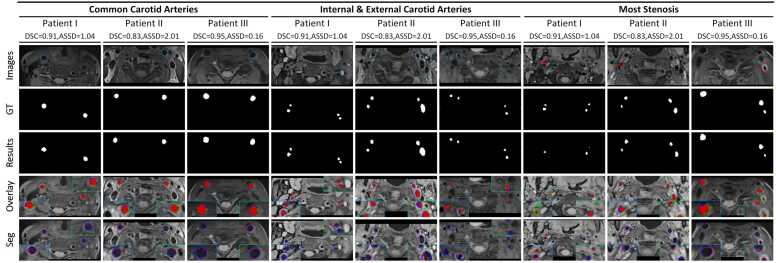
This is the 2D visualization of carotid artery segmentation results. Red, green, and blue areas are denoted as complete overlap, under-segmentation, and over-segmentation, respectively. Red and blue lines represent boundaries of ground truth and segmentation results. The blue dotted line and red arrow indicate the division area and the most stenosis area, respectively. Patient I, II, and III represent patients of mild, moderate, and severe stenosis, respectively. *2D* two-dimensional

**Fig. 8:**
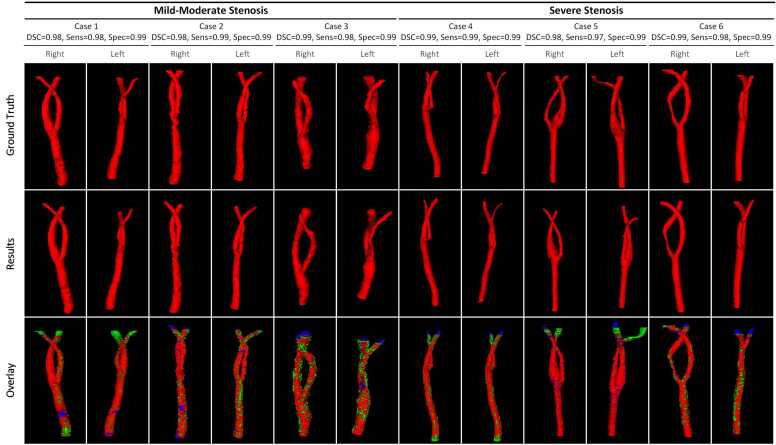
This is the 3D visualization of carotid artery segmentation results where “Case 1″, “Case 2″, and “Case 3″ are patients with mild-moderate stenosis of arteries, “Case 4″, “Case 5″, and “Case 6″ are patients with severe stenosis of arteries. Green, and blue pixel points are denoted as under-segmentation and over-segmentation, respectively. *3D* three-dimensional

As depicted in Table 3, the models trained for carotid artery segmentation demonstrated consistent performance during cross-validation. Corresponding high values of Dice similarity coefficient (DSC), intersection over union (IOU), Sens, and Spec demonstrate the excellent performance of the proposed carotid artery segmentation architecture. The lower values of relative volume error (RVE), average symmetric surface distance (ASSD), and the 95% Hausdorff distance (HD95) further support its precision. The results from the external validation set confirm the high generalization performance and robustness of the proposed architecture. The segmentation results of the proposed architecture on the external validation set were visualized from both 2D and 3D perspectives (Fig. 6), as shown in Figs. 7 and 8.

**Table 3:** Performance of carotid artery segmentation on the training-validation set, independent test set, and external validation set

Dataset	DSC	IOU	RVE	Sens	ASSD	HD95
*Training-validation set*						
Fold 0	0.98 ± 0.02	0.96 ± 0.03	0.01 ± 0.01	0.98 ± 0.02	0.12 ± 0.16	1.19 ± 2.33
Fold 1	0.98 ± 0.02	0.95 ± 0.03	0.03 ± 0.02	0.97 ± 0.03	0.18 ± 0.20	1.71 ± 2.98
Fold 2	0.98 ± 0.03	0.95 ± 0.05	0.03 ± 0.04	0.98 ± 0.02	0.23 ± 0.49	2.52 ± 7.01
Fold 3	0.98 ± 0.02	0.96 ± 0.03	0.02 ± 0.03	0.98 ± 0.02	0.16 ± 0.24	1.64 ± 3.65
Fold 4	0.98 ± 0.02	0.96 ± 0.03	0.02 ± 0.03	0.98 ± 0.02	0.17 ± 0.23	1.69 ± 3.45
Ensemble	0.98 ± 0.02	0.96 ± 0.04	0.02 ± 0.03	0.98 ± 0.02	0.17 ± 0.29	1.75 ± 4.21
*Independent testing set*						
Fold 0	0.96 ± 0.02	0.93 ± 0.04	0.05 ± 0.04	0.94 ± 0.04	1.05 ± 3.15	11.94 ± 35.83
Fold 1	0.97 ± 0.02	0.93 ± 0.03	0.05 ± 0.04	0.94 ± 0.04	0.41 ± 0.43	4.81 ± 6.04
Fold 2	0.96 ± 0.02	0.93 ± 0.04	0.05 ± 0.04	0.94 ± 0.04	0.49 ± 0.49	5.46 ± 6.41
Fold 3	0.96 ± 0.02	0.93 ± 0.04	0.05 ± 0.03	0.94 ± 0.04	0.47 ± 0.51	5.54 ± 8.13
Fold 4	0.96 ± 0.02	0.93 ± 0.04	0.05 ± 0.04	0.94 ± 0.04	0.55 ± 0.65	5.98 ± 8.21
Ensemble	0.97 ± 0.02	0.95 ± 0.04	0.05 ± 0.04	0.95 ± 0.04	0.36 ± 0.45	4.47 ± 6.29
*External validation set*						
Fold 0	0.94 ± 0.05	0.90 ± 0.07	0.06 ± 0.05	0.92 ± 0.05	0.80 ± 1.36	8.41 ± 12.63
Fold 1	0.94 ± 0.05	0.88 ± 0.08	0.06 ± 0.04	0.92 ± 0.06	0.95 ± 1.35	10.46 ± 14.60
Fold 2	0.94 ± 0.05	0.88 ± 0.08	0.07 ± 0.06	0.92 ± 0.05	0.78 ± 0.96	8.82 ± 11.32
Fold 3	0.94 ± 0.05	0.89 ± 0.07	0.06 ± 0.04	0.92 ± 0.05	0.68 ± 0.91	7.24 ± 8.81
Fold 4	0.93 ± 0.07	0.87 ± 0.10	0.08 ± 0.08	0.92 ± 0.05	1.01 ± 1.48	11.09 ± 15.13
Ensemble^a^	0.95 ± 0.05	0.91 ± 0.07	0.06 ± 0.04	0.93 ± 0.05	0.70 ± 1.15	7.91 ± 11.31

a *DSC* dice similarity coefficient, *IOU* intersection over union, *RVE* relative volume error, *Sens* sensitivity, *ASSD* average symmetric surface distance, *HD95* 95% Hausdorff Distance. All metrics are presented as "means ± standard deviation."

To evaluate the agreement between the ground truths and segmentation volumes, correlation plots were visualized in Fig. 6a, and concordance correlation coefficients (CCC) were calculated. The CCC values for the training-validation, independence test, and external validation sets were 0.985 (95% CI: 0.981–0.987), 0.979 (95% CI: 0.963–0.984), and 0.963 (95% CI: 0.944–0.992), respectively. These results indicate a high level of agreement between the ground truths and segmentation volumes, further validating the segmentation performance.

Moreover, to validate the generalization performance of the proposed architecture, 18 additional subgroup analyses have been conducted on both the independent test set and external validation set. Specifically, quantitative evaluations of segmentation performance across different stenosis severities and across three scanners have been performed, including entire extracranial carotid artery segmentation and slice-based stenotic region segmentation. For each subgroup, the CCC values for the segmentation results versus the ground truth have been included. The results, summarized in Table 4, demonstrated consistent performance across three scanners and stenosis severities, thereby confirming the robustness and reliability of the proposed architecture. These CCC values ranged from 0.90 to 0.98, further supporting the reliability and consistency of the model across different conditions.

**Table 4:** Subgroup analysis of segmentation performance across different stenosis severities and scanners

			Components			DSC	IOU	RVE	Sens	ASSD	HD95	CCC
						Independent testing set (Ensemble of folds)						
MS	SS	NR	ScI	ScII	ScIII							
*✓*	*✓*	*✓*	*✓*	*✓*	*✓*	0.97 ± 0.02	0.95 ± 0.04	0.05 ± 0.04	0.95 ± 0.04	0.36 ± 0.45	4.47 ± 6.29	0.98 [0.96–0.98]
*✓*	*✓*		*✓*	*✓*	*✓*	0.97 ± 0.02	0.95 ± 0.03	0.05 ± 0.03	0.95 ± 0.03	0.34 ± 0.40	4.08 ± 5.72	0.98 [0.97–0.98]
*✓*			*✓*	*✓*	*✓*	0.97 ± 0.02	0.94 ± 0.04	0.06 ± 0.04	0.94 ± 0.04	0.43 ± 0.56	5.41 ± 7.27	0.98 [0.94–0.99]
	*✓*		*✓*	*✓*	*✓*	0.98 ± 0.02	0.95 ± 0.03	0.05 ± 0.03	0.95 ± 0.03	0.30 ± 0.33	3.64 ± 5.08	0.98 [0.97–0.98]
*✓*	*✓*	*✓*	*✓*			0.98 ± 0.01	0.95 ± 0.03	0.05 ± 0.03	0.95 ± 0.03	0.34 ± 0.32	4.41 ± 5.38	0.97 [0.93–0.99]
*✓*	*✓*		*✓*			0.98 ± 0.02	0.96 ± 0.03	0.04 ± 0.03	0.96 ± 0.03	0.21 ± 0.18	2.43 ± 3.18	0.97 [0.95–0.99]
*✓*	*✓*	*✓*		*✓*		0.97 ± 0.02	0.95 ± 0.04	0.05 ± 0.04	0.95 ± 0.04	0.44 ± 0.58	5.42 ± 7.73	0.98 [0.95–0.99]
*✓*	*✓*			*✓*		0.97 ± 0.02	0.95 ± 0.04	0.05 ± 0.04	0.95 ± 0.04	0.37 ± 0.45	4.47 ± 6.14	0.98 [0.95–0.98]
*✓*	*✓*	*✓*			*✓*	0.97 ± 0.02	0.95 ± 0.03	0.05 ± 0.03	0.95 ± 0.03	0.26 ± 0.20	2.99 ± 3.57	0.97 [0.93–0.99]
*✓*	*✓*				*✓*	0.98 ± 0.02	0.95 ± 0.03	0.05 ± 0.03	0.95 ± 0.03	0.38 ± 0.43	4.66 ± 6.35	0.98 [0.96–0.99]

						External validation set (Ensemble of folds)						
MS	SS	NR	ScI	ScII	ScIII							
*✓*	*✓*	*✓*	*✓*	*✓*	*✓*	0.95 ± 0.04	0.91 ± 0.07	0.05 ± 0.03	0.93 ± 0.05	0.70 ± 1.15	7.90 ± 11.30	0.96 [0.94–0.99]
*✓*	*✓*		*✓*	*✓*	*✓*	0.94 ± 0.07	0.89 ± 0.10	0.09 ± 0.07	0.90 ± 0.08	0.80 ± 1.02	9.05 ± 10.37	0.94 [0.91–0.95]
*✓*			*✓*	*✓*	*✓*	0.94 ± 0.04	0.89 ± 0.07	0.10 ± 0.07	0.90 ± 0.07	0.68 ± 0.51	8.29 ± 5.61	0.97 [0.95–0.98]
	*✓*		*✓*	*✓*	*✓*	0.93 ± 0.07	0.88 ± 0.11	0.09 ± 0.08	0.89 ± 0.09	0.87 ± 1.15	9.59 ± 11.70	0.90 [0.85–0.93]
*✓*	*✓*	*✓*	*✓*			0.97 ± 0.01	0.95 ± 0.02	0.04 ± 0.02	0.95 ± 0.02	0.29 ± 0.36	2.82 ± 2.37	0.97 [0.92–0.99]
*✓*	*✓*		*✓*			0.96 ± 0.03	0.93 ± 0.06	0.07 ± 0.06	0.93 ± 0.06	0.40 ± 0.49	4.69 ± 6.01	0.99 [0.97–0.99]
*✓*	*✓*	*✓*		*✓*		0.95 ± 0.02	0.90 ± 0.04	0.06 ± 0.03	0.93 ± 0.04	0.95 ± 1.16	12.47 ± 17.03	0.92 [0.82–0.97]
*✓*	*✓*			*✓*		0.92 ± 0.05	0.86 ± 0.08	0.10 ± 0.06	0.88 ± 0.06	1.32 ± 1.43	17.09 ± 19.70	0.88 [0.57–0.96]
*✓*	*✓*	*✓*			*✓*	0.94 ± 0.05	0.90 ± 0.08	0.06 ± 0.04	0.92 ± 0.05	0.73 ± 1.24	8.08 ± 10.82	0.97 [0.95–0.98]
*✓*	*✓*				*✓*	0.93 ± 0.07	0.88 ± 0.11	0.10 ± 0.08	0.89 ± 0.09	0.81 ± 0.98	8.66 ± 7.59	0.93 [0.90–0.96]

*MS* moderate stenosis, *SS* severe stenosis, and *NR* normal regions within lumen, respectively. Moreover, ‘ScI’, ‘ScII’, and ‘ScIII’ refer to the Philips Ingenia (ScI), GE Discovery MR750W (ScII), and Siemens Magnetom Prisma (ScIII) acquisition platform, respectively.

### 4.4 Performance of artery stenosis quantitative evaluation

**Fig. 9:**
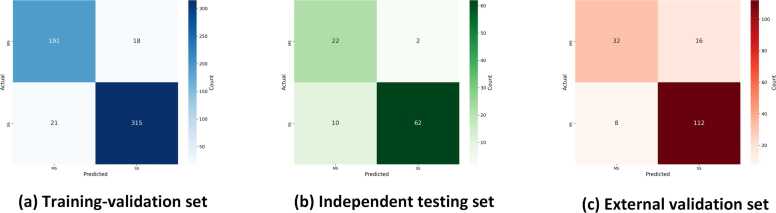
The figure shows the confusion matrix of the stenosis quantitative evaluation module on three datasets. (a) Training-validation set: the module shows high classification accuracy. (b) Independent test set: on the independent test set, the module still maintains better performance, which shows a strong generalization ability. (c) External validation set: On the external validation set, the module also performs well, which verifies the validity of the module in practical applications

This subsection evaluates the performance of the stenosis quantitative evaluation module in detail. Among the training-validation set and independent test set, 641 lesion-wise diseased vessels were diagnosed by radiologists using DSA. The results on the training-validation set are shown in Table 5. The five-fold cross-validation demonstrated outstanding performances, with accuracy (Acc) ranging from 0.87 to 0.96, F1-Score ranging from 0.88 to 0.97, sensitivity (Sens) ranging from 0.86 to 0.99, Precision ranging from 0.89 to 0.96, specificity (Spec) ranging from 0.87 to 0.93, and the AUC ranging from 0.87 to 0.95. The confusion matrix for the ensemble model is displayed in Fig. 9a. For the ensemble of folds on the independent test set, Acc, F1-Score, Sens, Precision, Spec, and AUC were 0.88, 0.91, 0.86, 0.97, 0.92, and 0.89, respectively. The corresponding confusion matrix is depicted in Fig. 9b.

**Table 5:** Performance of stenosis quantitative evaluation on the training-validation set, independent test set, and external validation set

Dataset	Acc	F1-Score	Sens	Precision	Spec	AUC
*Training-validation set*						
Fold 0	0.93	0.93	0.92	0.95	0.93	0.93
Fold 1	0.95	0.96	0.97	0.96	0.93	0.95
Fold 2	0.93	0.95	0.93	0.96	0.92	0.93
Fold 3	0.87	0.88	0.86	0.89	0.87	0.87
Fold 4	0.96	0.97	0.99	0.96	0.92	0.95
Ensemble	0.93	0.94	0.94	0.95	0.91	0.93
*Independent testing set*						
Fold 0	0.85	0.90	0.92	0.89	0.67	0.79
Fold 1	0.83	0.90	0.97	0.83	0.42	0.69
Fold 2	0.88	0.92	0.97	0.88	0.58	0.78
Fold 3	0.85	0.91	0.97	0.85	0.50	0.74
Fold 4	0.83	0.89	0.92	0.87	0.58	0.75
Ensemble	0.88	0.91	0.86	0.97	0.92	0.89
*External validation set*						
Fold 0	0.81	0.87	0.93	0.82	0.50	0.72
Fold 1	0.81	0.87	0.87	0.87	0.67	0.77
Fold 2	0.76	0.81	0.73	0.92	0.83	0.78
Fold 3	0.71	0.77	0.67	0.91	0.83	0.75
Fold 4	0.86	0.91	0.87	0.83	0.50	0.75
Ensemble	0.86	0.90	0.93	0.88	0.67	0.80

The high values of these metrics in Table 5 indicate excellent performance on the external validation set. Furthermore, the high sensitivity and specificity demonstrate low rates of missed and misdiagnosis, respectively. The corresponding confusion matrix for the ensemble model on the external validation set is depicted in Fig. 9c. These results further confirm the high generalization performance and robustness of the proposed module.

### 4.5 Comparison with ablation and benchmark results

As demonstrated in Table 6, the proposed architecture outperformed all ablation and benchmark models on segmentation tasks, which achieved the highest DSC, IOU, RVE, and Sens values. It also recorded the fourth-highest Spec, with an extremely small gap, and the lowest RVE, ASSD, and HD95 on the training-validation set. On both the independent test set and external validation set, this work outperformed the benchmark experiments across all metrics. Specifically, the agreement between ground truths and segmentation results from the ablation experiment, CCC is depicted in Fig. 6b.

**Table 6:** Comparison of segmentation performance between the proposed module, ablation models, and benchmark models on the training-validation set, independent test set, and external validation set

Dataset	Model	DSC	IOU	RVE	Sens	ASSD	HD95
				*Training-validation set (ensemble of folds)*			
Benchmark	3D-UNet	0.74 ± 0.23	0.62 ± 0.21	0.24 ± 0.21	0.74 ± 0.23	6.99 ± 16.25	42.45 ± 51.39
	U2Net	0.77 ± 0.19	0.66 ± 0.21	0.30 ± 0.32	0.71 ± 0.22	4.92 ± 11.85	25.46 ± 40.83
Ablation study	w/o LC	0.94 ± 0.03	0.88 ± 0.05	0.05 ± 0.05	0.93 ± 0.04	0.69 ± 0.70	6.51 ± 9.17
	Proposed (ours)	**0.98** ± **0.02**	**0.96** ± **0.04**	**0.02** ± **0.03**	**0.98** ± **0.02**	**0.17** ± **0.29**	**1.75** ± **4.21**
	P-value	< 0.01*	< 0.01*	< 0.01*	< 0.01*	< 0.01*	< 0.01*
				*Independent testing set (ensemble of folds)*			
Benchmark	3D-UNet	0.69 ± 0.20	0.55 ± 0.18	0.28 ± 0.24	0.67 ± 0.25	3.70 ± 5.75	21.86 ± 33.15
	U2Net	0.75 ± 0.20	0.64 ± 0.22	0.33 ± 0.33	0.69 ± 0.22	5.90 ± 11.33	31.12 ± 43.27
Ablation study	w/o LC	0.95 ± 0.02	0.91 ± 0.03	**0.04** ± **0.03**	0.93 ± 0.04	0.62 ± 0.50	6.72 ± 6.91
	Proposed (ours)	**0.97** ± **0.02**	**0.95** ± **0.04**	0.05 ± 0.04	**0.95** ± **0.04**	**0.36** ± **0.45**	**4.47** ± **6.29**
	P-value	< 0.01*	< 0.01*	0.08	< 0.01*	< 0.01*	< 0.01*
				*External validation set (ensemble of folds)*			
Benchmark	3D-UNet	0.64 ± 0.14	0.48 ± 0.14	0.75 ± 1.01	0.64 ± 0.19	3.90 ± 5.03	17.69 ± 20.33
	U2Net	0.74 ± 0.20	0.62 ± 0.21	0.34 ± 0.36	0.68 ± 0.23	6.42 ± 14.96	29.63 ± 43.01
Ablation study	w/o LC	0.94 ± 0.02	0.88 ± 0.04	0.07 ± 0.05	0.91 ± 0.05	1.10 ± 1.05	15.14 ± 16.88
	Proposed (ours)	**0.95** ± **0.05**	**0.91** ± **0.07**	**0.06** ± **0.04**	**0.93** ± **0.05**	**0.70** ± **1.15**	**7.91** ± **11.31**
	P-value	< 0.01*	< 0.01*	0.89	< 0.01*	< 0.01*	0.06

Bold values are the optimal values in the corresponding index results. They reflect the outperformance of the proposed method. The asterisk (*) indicates the statistically significant difference (P < 0.05) in the comparison and w/o = without, *DSC* dice similarity coefficient, *IOU* intersection over union, *RVE* relative volume error, *Sens* sensitivity, *ASSD* average symmetric surface distance, *HD95* 95% Hausdorff Distance.

**Fig. 10:**
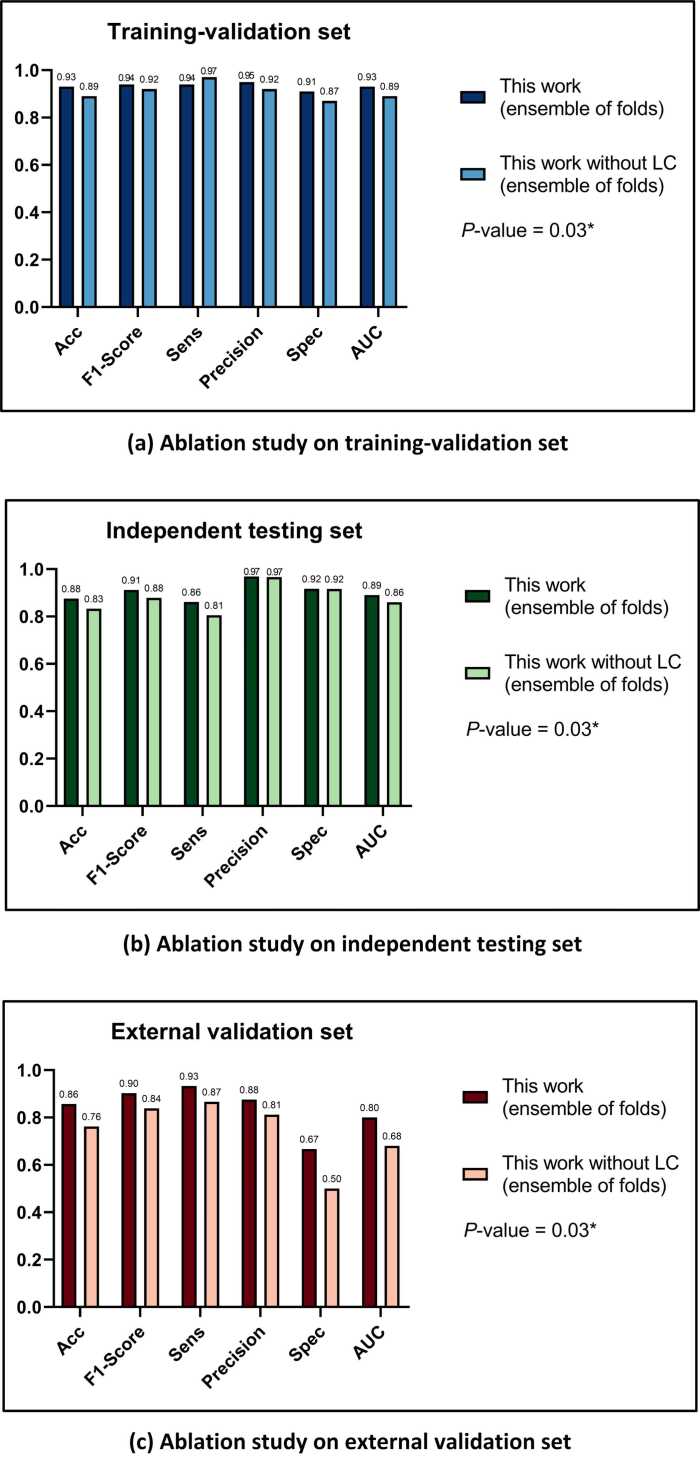
This figure compares the performance of stenosis quantitative evaluation in ablation experiment in histograms. The results on (a) Training-validation set, (b) Independent test set, and (c) External validation set demonstrate the proposed work had a much better performance than the work without LC. *LC* localization model, *Acc* accuracy, *Sens* sensitivity, *Spec* specificity, *AUC* area under the curve

In comparison to the performance of stenosis quantitative evaluation in the ablation experiment, this work demonstrated superior performance across all metrics, as illustrated in the histogram in Fig. 10. These results not only demonstrate the effectiveness of the proposed LC module but also highlight the enhanced segmentation performance and robustness achieved by incorporating the residual structure into the network.

These results not only demonstrate the effectiveness of the proposed LC module but also highlight the enhanced segmentation performance and robustness achieved by incorporating the residual structure into the network. Specifically, the LC module accurately identifies the carotid artery, allowing the subsequent segmentation steps to focus on the specific region of interest (ROI), thereby eliminating interference from the surrounding regions and improving overall performance.

## 5 Discussion

This study has presented a multi-stage deep learning architecture for the automated segmentation and quantitative evaluation of stenosis in extracranial carotid arteries using HR-MRI scans. The proposed architecture demonstrated exceptional performance on the multicenter datasets of four tertiary hospitals. By minimizing inter-observer variability, this architecture offers a reliable and efficient tool for diagnosing head and neck atherosclerotic disease and assessing stroke risk in clinical practice.

The proposed architecture contained three modules: the LC, the automatic segmentation module, and the stenosis quantitative evaluation module. The LC identified the ROI from a 2D perspective, ensuring the inclusion of the entire extracranial portion of the carotid arteries. The automatic segmentation module performs precise 3D segmentation of the carotid arteries. Lastly, the stenosis quantitative evaluation module ensures the classification of the degree of stenosis within the segmented carotid artery.

Specifically, the segmentation module was trained on a dataset labeled by experienced radiologists, while the stenosis degrees, as diagnosed by DSA, served as the ground truth for the stenosis quantitative evaluation module.

The complex structure of carotid arteries poses significant challenges for segmentation algorithms. Maintaining a consistent and continuous 3D shape is difficult, especially given that carotid plaque volume occupies less than 0.1% of the MR image space.

To address these challenges, the LC was designed to facilitate the localization of ROI. Building upon this, an end-to-end architecture with residual connections in the encoder was employed for segmentation within ROI. The modified 3D U-net aimed to enhance training efficiency and segmentation accuracy while mitigating the issue of vanishing gradients in deep networks.

The results of ablation experiments further validated the effectiveness of the LC, which improved key performance metrics on both the independent test set and external validation set. Subsequently, the stenosis quantitative evaluation module was implemented to develop a fully automated and efficient workflow for stenosis diagnosis. The segmentation results were initially straightened to correct for lumen distortion. Arterial stenosis was then quantitatively evaluated according to the NASCET criteria. Additionally, the sum of pixel volumes for each slice was calculated to visualize suspected stenotic regions in the ICA and CCA, aiding in treatment planning.

**Fig. 11:**
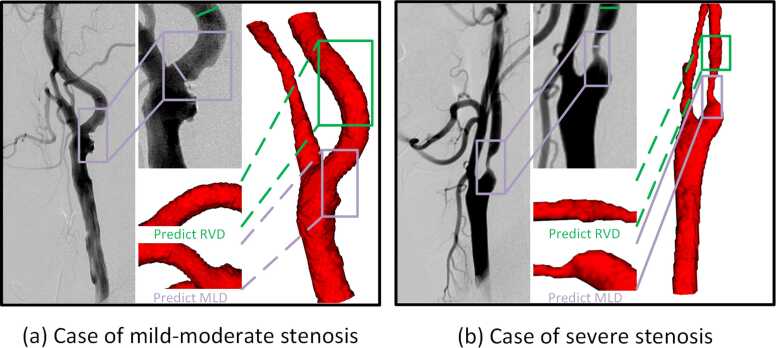
Comparative analysis of HR-MRI-based carotid segmentations and the carotid arteries visualized by DSA on (a) mild-moderate stenosis lesion-wise case and (b) severe stenosis lesion-wise case. The inset is a zoom of the graph near the selected region. Purple and green boxes indicate MLD and RVD, respectively. The dashed and solid lines indicate the predicted position based on the proposed module and the real position observed by DSA, respectively. *HR-MRI* High-resolution magnetic resonance imaging, *DSA* digital subtraction angiography, *MLD* minimum lumen diameter, *RVD* reference vessel diameter

Before training the overall architecture, image preprocessing and augmentation were performed to ensure data consistency and improve model performance. Specifically, the images were resampled by adjusting the median voxel spacing, which ensured consistency throughout the dataset and enhanced model training and generalization. Subsequently, Z-Score-Normalization was employed to normalize the image data by subtracting the mean and dividing it by the standard deviation, thereby aligning the data distribution with the standard normal distribution to improve model training and generalization. Furthermore, the labels of each layer were inflated according to a designed formula to ensure complete coverage of the extracranial carotid artery region. Fig. 11.

**Fig. 12:**
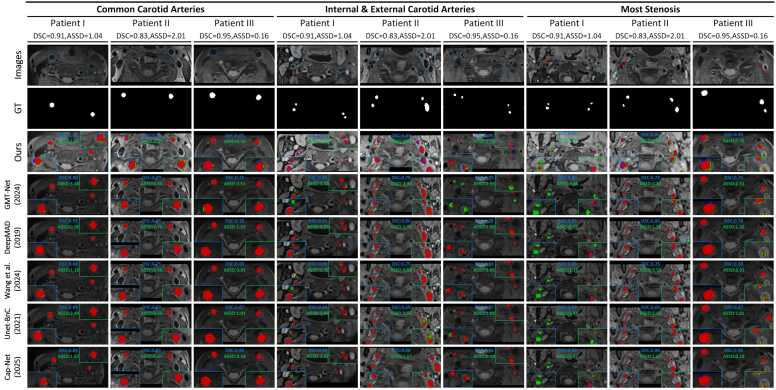
Qualitative comparison of carotid artery segmentation results between the proposed method and five SOTA models. Red, green, and blue areas are denoted as complete overlap, under-segmentation, and over-segmentation, respectively. Patient I, II, and III represent patients of mild, moderate, and severe stenosis, respectively. *SOTA* state-of-the-art

Compared with previous studies, the proposed architecture demonstrated superior performance, as depicted in Table 7. In the segmentation task, our architecture achieved a higher value of DSC while maintaining a relatively low ASSD. Moreover, additional experiments have been conducted to directly compare our approach with five state-of-the-art (SOTA) networks on both independent testing and external validation datasets. These models were selected based on their established success in related segmentation tasks, providing a robust basis for comparison. All methods were retrained and evaluated under the same experimental settings and datasets to ensure a fair comparison, and the key parameter settings have been provided in Supplementary Note 5. Compared with previous studies, the proposed architecture demonstrated superior performance, as depicted in Table 7. In the segmentation task, our architecture achieved a higher value of DSC while maintaining a relatively low ASSD. The segmentation results of Unet-BnC (Zhu et al. [22]), Wang et al. [26], DeepMAD (Wu et al. [39]), GMT-Net (Wu et al. [19]), and CAP-Net (Luo et al. [44]) are summarized in Table 8, our method consistently outperformed these approaches on both the independent testing set and the external validation set, achieving higher DSC and IoU while maintaining competitive ASSD and HD95 values. Notably, on the external validation set, our method achieved a DSC of 0.95 ± 0.04, which is superior to GMT-Net (0.91 ± 0.07) and CAP-Net (0.91 ± 0.07). In addition to quantitative metrics, a qualitative comparison was performed to visually assess segmentation performance between our work and SOTA in Fig. 12. As shown in column 7 of Fig. 12, the proposed method demonstrates closer agreement with the manual reference across different stenosis severities. In particular, our approach provides more complete lumen preservation and better boundary continuity in ICA, ECA, and the most stenotic segment, effectively reducing under-segmentation artifacts commonly observed in the comparison networks. These visual comparisons further confirm the robustness and clinical applicability of the proposed model. Furthermore, the intra-class correlation coefficient (ICC) was utilized to quantitatively assess the consistency between HR-MRI-based carotid segmentation results and the carotid arteries shown by DSA. Three experienced radiologists independently evaluated the concordance across 168 cases from the external validation set. Each radiologist measured the diameter of the lumen as segmented by the model and as depicted by DSA in three transverse view positions (bifurcation of the common carotid artery, beginning of the ICA, and the narrowest point of the ICA). The mean value of ICC was 0.926 (95% CI: 0.886–0.942), which demonstrated a high concordance in morphology between the HR-MRI-based carotid segmentations and the DSA-derived ones. As depicted in Fig. 11, the segmentation results in mild-moderate and severe stenosis were visualized and compared with their corresponding DSA images. Regarding the diagnosis module, our workflow outperformed the methods of Qiu et al. [40], Wulamu et al. [7], Chung et al. [41], Khedmati et al. [42], and Chang et al. [43] in terms of Precision and Sens, reflecting high-performance diagnostic performance and a low rate of missed diagnosis. As shown by the experimental results, the proposed architecture demonstrated high consistency with manual segmentation and DSA diagnostic criteria, which affirmed its clinical utility in enhancing the accuracy of vascular assessments.

**Table 7:** Comparison of the proposed work with previous studies in terms of segmentation performance and stenosis quantitative evaluation performance

Methods	Segmentation		Methods	Evaluation	
	DSC*↑*	ASSD		Precision*↑*	Sens
Zhu et al. [22]	0.8453	0.7021	Qiu et al. [40]	0.7110	0.8650
Wang et al. [26]	0.8459	**0.1127**	Wulamu et al. [7]	0.7882	0.9114
Wu et al. [39] (AIM-HIGH Trail test)	0.9475	-	Chung et al. [41]	0.8100	0.8300
Wu et al. [39] (CAREII test)	0.9594	-	Abolfazl et al. [42]	-	0.8900
Wu et al. [19]	0.9677	-	Chang et al. [43]	0.9650	0.8720
This work (Independent testing set)	**0.9737**	0.3634	This work (Independent testing set)	**0.9688**	0.8611
This work (External validation set)	0.9531	0.7017	This work (External validation set)	0.8750	**0.9333**

Bold values are the optimal values in the corresponding index results. They reflect the outperformance of the proposed method. The delineate (-) indicates the corresponding evaluation indicators had not been provided in the original text. *DSC* dice similarity coefficient, *ASSD* average symmetric surface distance.

**Table 8:** Comparison of segmentation performance between the proposed method and previous studies on the independent test set and the external validation set

Method	DSC	IOU	RVE	Sens	ASSD	HD95
			*Independent testing set (ensemble of folds)*			
GMT-Net [19]	0.96 ± 0.02	0.93 ± 0.04	0.03 ± 0.03	0.96 ± 0.03	0.33 ± 0.39	2.60 ± 5.53
DeepMAD [39]	0.95 ± 0.02	0.91 ± 0.04	0.02 ± 0.02	0.95 ± 0.02	0.34 ± 0.34	2.61 ± 5.87
Wang et al. [26]	0.89 ± 0.02	0.87 ± 0.03	0.02 ± 0.02	0.90 ± 0.01	0.25 ± 0.20	1.40 ± 2.52
Unet-BnC [22]	0.88 ± 0.03	0.85 ± 0.05	0.03 ± 0.04	0.88 ± 0.02	0.38 ± 0.49	2.61 ± 5.63
CAP-Net [44]	0.93 ± 0.02	0.91 ± 0.03	0.02 ± 0.03	0.94 ± 0.01	0.20 ± 0.23	1.27 ± 2.55
**This work**	**0.97** ± **0.02**	**0.95** ± **0.04**	**0.05** ± **0.04**	**0.95** ± **0.04**	**0.36** ± **0.45**	**4.47** ± **6.29**
			*External validation set (ensemble of folds)*			
GMT-Net [19]	0.80 ± 0.07	0.67 ± 0.10	0.12 ± 0.11	0.80 ± 0.09	3.50 ± 2.04	31.13 ± 15.45
DeepMAD [39]	0.91 ± 0.07	0.85 ± 0.11	0.09 ± 0.09	0.91 ± 0.05	1.22 ± 1.60	13.54 ± 16.12
Wang et al. [26]	0.87 ± 0.06	0.78 ± 0.09	0.11 ± 0.10	0.86 ± 0.08	1.65 ± 1.30	17.63 ± 16.18
Unet-BnC [22]	0.87 ± 0.04	0.77 ± 0.07	0.11 ± 0.08	0.84 ± 0.07	2.15 ± 1.23	23.28 ± 16.06
CAP-Net [44]	0.91 ± 0.07	0.83 ± 0.10	0.07 ± 0.07	0.90 ± 0.08	1.42 ± 1.98	13.88 ± 18.31
**This work**	**0.95** ± **0.04**	**0.91** ± **0.07**	**0.05** ± **0.03**	**0.93** ± **0.05**	**0.70** ± **1.15**	**7.90** ± **11.30**

Bold values are the optimal values in the corresponding index results. *DSC* dice similarity coefficient, *IOU* intersection over union, *RVE* relative volume error, *Sens* sensitivity, *ASSD* average symmetric surface distance, *HD95* 95% Hausdorff Distance. All metrics are presented as "means ± standard deviation."

## 6 Limitations

Despite the positive findings, several limitations need to be considered. The generalizability of the proposed architecture should be further validated on a more diverse and comprehensive dataset to ensure wider applicability, especially in cases such as carotid stenosis caused by arteritis, arterial dissection, and other diseases. The dataset currently used may not fully represent all the anatomical and pathological variations encountered in clinical practice. The missed segmentation observed in Patient I (Fig. 7), where one artery in the most stenotic region was not captured, is a key example of such limitations. To address the issue of missed segmentation, expanding the dataset to include a wider range of cases will improve the model’s robustness and generalization capabilities. Furthermore, we will standardize the annotation process and implement stricter controls over the annotation workflow to ensure that all critical regions, including stenotic areas, are consistently captured. This will be part of our ongoing efforts to improve the model’s performance and robustness. Additionally, the sequential two-phase architecture requires significant computational resources. Future modifications should focus on optimizing the inference process for faster and more efficient performance. Following that, while the diagnostic module performs well overall, it demonstrates limitations in diagnosing lesions with mild to moderate stenosis (with error rates of 33.3% and 6% for mild-moderate and severe stenosis, respectively), indicating the need for further optimization to enhance robustness and diagnostic accuracy. Specifically, the inclusion of adjacent structures, such as the jugular vein, in the ROI during the coarse localization stage can lead to incorrect measurements of the lumen and reference vessel diameters, which are critical for accurate stenosis quantification. In future work, we plan to refine the coarse localization module to ensure better separation of the carotid artery from surrounding structures. This will include incorporating more precise anatomical constraints to prevent the inclusion of non-arterial tissues, thereby improving segmentation accuracy and stenosis evaluation. Finally, although the current study primarily focuses on stenosis quantification using HR-MRI, we recognize that HR-MRI has additional potential to assess plaque characteristics such as plaque burden and vulnerability. Future research will expand our analysis to incorporate vessel wall segmentation, as well as detailed assessments of plaque burden and vulnerability, to fully leverage HR-MRI’s capabilities and enhance the clinical relevance of our findings.

## 7 Conclusion

In conclusion, this study presented a multi-stage deep learning architecture for the automated segmentation and quantitative evaluation of stenosis in extracranial carotid arteries using HR-MRI scans. The proposed architecture demonstrated outstanding performance across multicenter datasets. Additionally, by minimizing inter-observer variability, this architecture offers a reliable and efficient tool for diagnosing head and neck atherosclerotic disease and assessing stroke risk in clinical practice.

## Author contributions

Wrote code and performed experiments: Z.Z. and X.L. Wrote the first draft of the manuscript: Z.Z., W.L. and X.C. Multicenter data collection and annotation: W.L, X.C., Z.Z., H.F, Q.L., Z.C., K.P. and D.G. Statistical analysis and data interpretation: Z.Z, W.L., X.C. and D.G. Provision of general research guidance: X.C. and D.G. All authors were involved in major revisions of the manuscript and read and approved the final version. Z.Z. and W.L. contributed equally to this work.

## Declaration of competing interests

The authors declare that they have no known competing financial interests or personal relationships that could have appeared to influence the work reported in this paper.
