# Supplementary material for: Multi-stage deep learning architecture for carotid artery segmentation and stenosis evaluation: comparative study with digital subtraction angiography
Source: J Cardiovasc Magn Reson. 2026 Jan 7;28(1):102683. doi: 10.1016/j.jocmr.2026.102683 (PMC13156810; doi:10.1016/j.jocmr.2026.102683)
Supplement: Supplementary file 1 — Supplementary material [file mmc1.pdf]

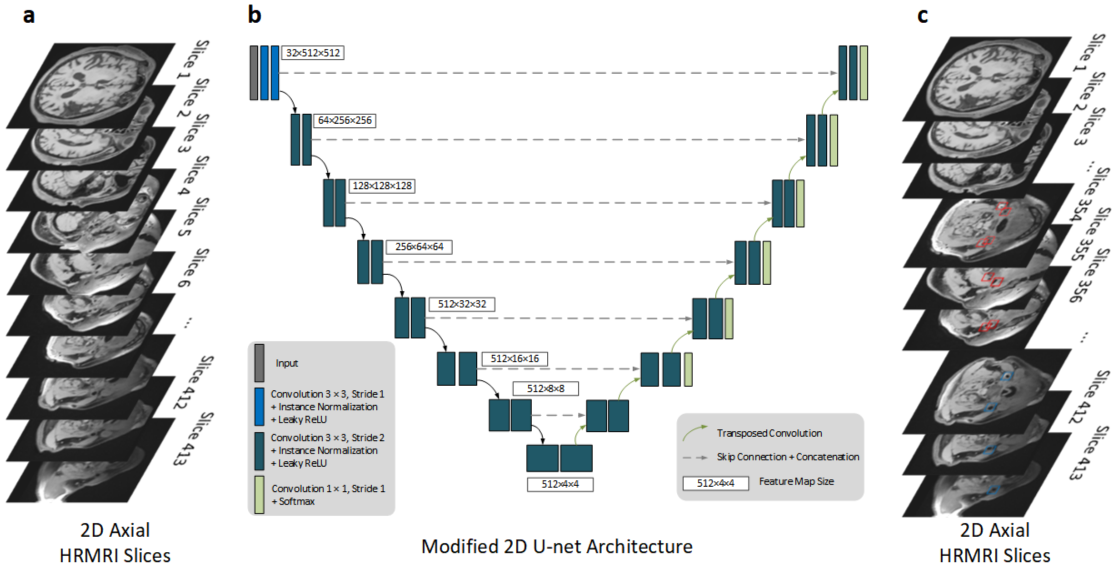

Supplementary Note 1 | This is the workflow of artery localization module, which consists of an encoder with 8 stages and a decoder with 7 stages. The 2D convolution operation with a normalization and an activation function is performed twice on each stage. Furthermore, the encoder and decode are connected via skip connections. The encoder comprises several stages, each containing two blocks: a convolution layer with a 3 × 3 kernel, followed by an instance normalization layer, and a leaky ReLU activation layer. Notably, downsampling is achieved between each layer of the encoder by a convolution operation with a stride of 2 (stride of 1 on the first stage). Based on this, the extraction and recovery of image features can be refined layer by layer, thus achieving high accuracy performance in image segmentation tasks. The decoder adopts a similar architecture, employing transposed convolution for upsampling the merged features that combine up-sampled outputs from the preceding layer and the skip-connected features from the corresponding encoder level. This design aims to effectively reduce the spatial dimensionality of the feature maps while maintaining important image information.

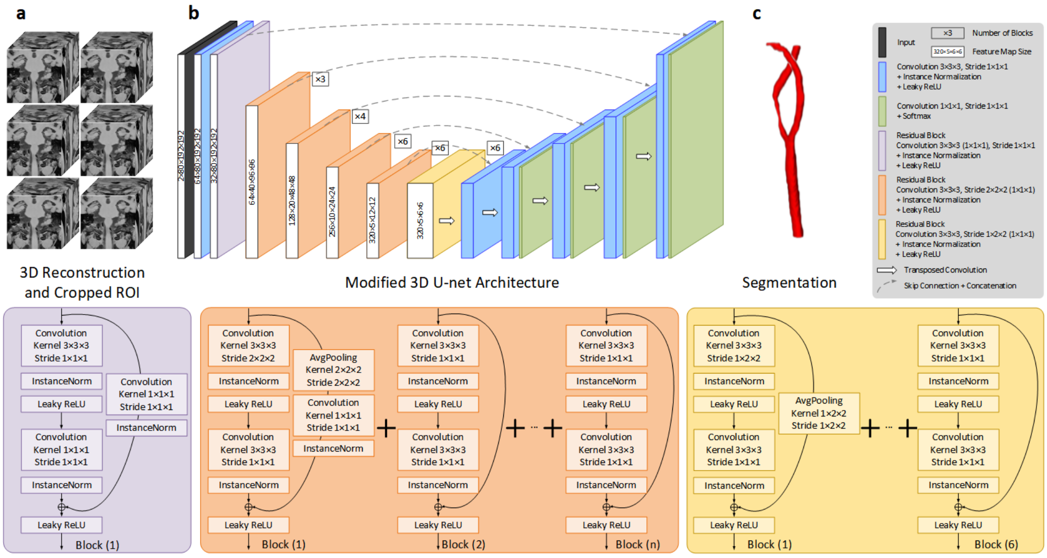

Supplementary Note 2 | This is the workflow of automatic segmentation module, which consists of a residual encoder with 6 stages and a decoder with 5 stages. The residual blocks which consisted of 3D convolution operation, normalization and activation function are performed twice on each stage. Furthermore, the average pooling is employed in the first block of the orange and yellow part. At the residual block of the highest resolution, the residual connection consisted of a Convolution operation (kernel of 1 × 1 × 1, stride of 1 × 1 × 1) and an InstanceNormalization operation. Following that, the residual connection of the first block was composed of an AvgPooling operation (kernel of 2 × 2 × 2, stride of 2 × 2 × 2), a Convolution operation (kernel of 1 × 1 × 1, stride of 1 × 1 × 1), and an InstanceNormalization operation. In the first block of the lowest resolution, an AvgPooling operation (kernel of 1 × 2 × 2, stride of 1 × 2 × 2) was employed to implement residual connection. This architecture enables the simultaneous of the lumens in both carotid arteries. Additionally, the number of feature mappings was set to 32, 64, 128, 256, 320, and 320 to accommodate computational resource constraints.

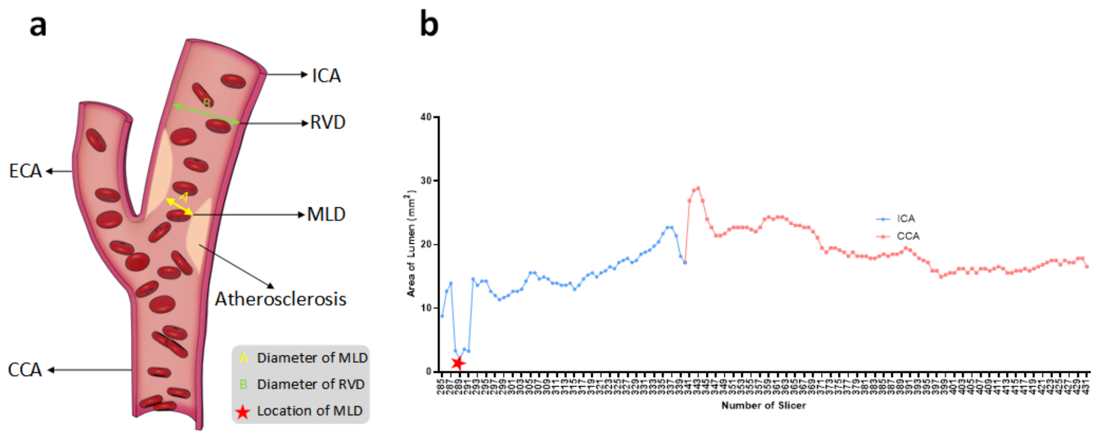

Extracranial Carotid Artery

Lumen Area Visualization

Supplementary Note 3 | (a) shows the locations of the RVD and the MLD at the stenotic region of the carotid artery. These measurements are essential for determining the degree of stenosis. (b) presents a graph illustrating the lumen area quantification process, where the horizontal axis represents slice number and the vertical axis shows the corresponding lumen area in terms of pixel volume. This approach aids in visualizing the entire extracranial carotid artery and identifying potential stenotic segments. The stenosis degree is calculated as (1-A/B)×100%, where A and B are donated as "Diameter of Minimum Lumen Diameter" and "Diameter of Reference Vessel Diameter", respectively. This module provided a systematic approach to detecting and evaluating arterial stenosis degree, leveraging advanced imaging and computational techniques to improve diagnostic workflows, reduce variability, and support timely clinical interventions. This process provides clinicians with a comprehensive view of the extracranial carotid arteries and allows for the identification of suspected stenotic areas.

Supplementary Note 4 | Evaluation metrics

To comprehensively quantify the performance of the proposed deep learning-enhanced architecture, this subsection details the evaluation metrics involved in the experiments. The metrics evaluate the segmentation results of the extracranial arteries and the lesion-wise diagnostic performance.

For the segmentation of the extracranial arteries, the performance of the combined model was evaluated as the following metrics:

(1) Dice similarity coefficient (DSC): This metric is used to evaluate the performance of the model by calculating the similarity between the segmentation results and the ground truths, as equation (2).

$$\mathrm{DSC}(Y_i, \widehat{Y_i}) = \frac{2|Y_i \cap \widehat{Y_i}|}{|Y_i| + |\widehat{Y_i}|} \tag{2}$$

where Y_i and $\widehat{Y_i}$ are the ground truth and segmentation results of the i-th sample, respectively. The closer the value of DSC is to 1, the better the segmentation effect of the model is.

(2) Intersection over union (IOU): This metric is used to evaluate the degree of overlap between the segmentation results and the ground truths, which can be formalized as equation (3):

$$\mathrm{IOU}(Y_i, \widehat{Y_i}) = \frac{|Y_i \cap \widehat{Y_i}|}{|Y_i \cup \widehat{Y_i}|} \tag{3}$$

The closer the value of IOU is to 1, the better the segmentation effect of the model is.

(3) Average symmetric surface distance (ASSD): This metric is a measure of the average surface distance between the segmentation results and the ground truths, which can be formalized as equation (4):

$$\mathrm{ASSD}(Y_i,\widehat{Y_i}) = \frac{1}{|S(Y_i)| + |S(\widehat{Y_i})|}\left(\sum_{y_j \in S(Y_i)} \min_{\widehat{y_j} \in S(\widehat{Y_i})} \left\|y_j - \widehat{y_j}\right\| + \sum_{\widehat{y_j} \in S(\widehat{Y_i})} \min_{y_j \in S(Y_i)} \left\|\widehat{y_j} - y_j\right\|\right) \quad (4)$$

where $S(Y_i)$ and $S(\widehat{Y_i})$ denote the set of surface voxels of Y_i and $\widehat{Y_i}$, respectively, and $||.||$ represents the Euclidean distance.

(4) 95% Hausdorff distance (HD95): This metric is a variant of Hausdorff distance (HD), which describes a measure of similarity between two sets of point sets. Here, we took the 95th percentile instead of the absolute maximum to avoid eliminating the effects of outliers. Its formula is shown as equation (5):

$$\mathrm{HD95}(Y_i,\widehat{Y_i}) = \max\left\{95^{th} \max_{y_j \in S(Y_i)}\left\{\min_{\widehat{y_j} \in S(\widehat{Y_i})}\left\|y_j - \widehat{y_j}\right\|\right\}, \max_{\widehat{y_j} \in S(\widehat{Y_i})}\left\{\min_{y_j \in S(Y_i)}\left\|\widehat{y_j} - y_j\right\|\right\}\right\} \quad (5)$$

(5) Relative volume error (RVE): This metric is used to measure the difference between the volume of the segmentation result of the algorithm and the volume of the actual labels, as equation (6):

$$\mathrm{RVE}(Y_i,\widehat{Y_i}) = \frac{|Y_i| - |\widehat{Y_i}|}{|Y_i|} \quad (6)$$

The smaller the value of RVE, the better the segmentation effect of the algorithm.

(6) Sensitivity (Sens): This metric measures the ability of the model to detect actual positive samples (e.g., lesions and specific anatomical structures), as equation (7):

$$\mathrm{Sens} = \frac{TP}{TP + FN} \quad (7)$$

where TP denotes true positives, and FN denotes false negatives. Higher Sens means better segmentation performance of the model.

(7) Specificity (Spec): This metric measures the ability of the model to correctly identify actual negative samples (e.g., normal tissue and lesion-free regions), as equation (8):

$$\mathrm{Spec} = \frac{TN}{TN + FP} \tag{8}$$

where TN denotes true negatives, and FP denotes false positives. The higher the Spec, the lower the possibility that the algorithm will misidentify normal regions as lesions.

Supplementary Note 5 | Key parameter settings of compared model

Method	Architecture	Learning Rate	Batch Size	Optimizer
GMT-Net	Shared encoder and separate decoders	0.0001	10	Adam (initial learning rate of 0.0001, decayed by 0.1 every 10 epochs)
DeepMAD	U-Net-based	0.0001	12	Adam
Wang et al.	Modified 3D U-Net	0.0001	8	SGD with momentum
Unet-BnC	U-Net-based	0.0001	16	Adam ($\beta1 = 0.9$ $\beta1 = 0.999$)
CAP-Net	Modified U-Net	0.0001	16	Adam
